# Abnormal behaviours relevant to neurodevelopmental disorders in Kirrel3-knockout mice

**DOI:** 10.1038/s41598-018-19844-7

**Published:** 2018-01-23

**Authors:** Tomoko Hisaoka, Tadasuke Komori, Toshio Kitamura, Yoshihiro Morikawa

**Affiliations:** 1 Department of Anatomy and Neurobiology, Wakayama Medical University, 811-1 Kimiidera, Wakayama, 641-8509 Japan; 2 Division of Cellular Therapy, Advanced Clinical Research Center, The Institute of Medical Science, The University of Tokyo, 4-6-1 Shirokanedai, Minato-ku, Tokyo, 108-8639 Japan

## Abstract

In the nervous system, Kirrel3 is involved in neuronal migration, axonal fasciculation, and synapse formation. Recently, genetic links have been reported between mutations in the KIRREL3 gene and increased risk of neurodevelopmental disorders, including autism spectrum disorder (ASD) and intellectual disability. To elucidate the causal relationship between KIRREL3 deficiency and behavioural abnormalities relevant to neurodevelopmental disorders, we generated global Kirrel3-knockout (Kirrel3^−/−^) mice and investigated the detailed behavioural phenotypes. In the three-chambered social approach test, Kirrel3^−/−^ mice displayed a significant preference for a mouse over a non-social object but no significant preference for a stranger mouse over a familiar mouse. Ultrasonic communications, including pup-to-mother calls, male-female courtship vocalisation and resident responses to intruder, were significantly impaired in Kirrel3^−/−^ mice. Significant increases in locomotor activity and repetitive rearing were also observed in Kirrel3^−/−^ mice. Furthermore, the performance of Kirrel3^−/−^ mice in the rotarod test was significantly better than that of wild-type mice. In the acoustic startle test, Kirrel3^−/−^ mice were significantly hypersensitive to acoustic stimuli. Anxiety-related behaviours and spatial or fear memory acquisition were normal in Kirrel3^−/−^ mice. These findings suggest that Kirrel3^−/−^ mice exhibit autistic-like behaviours, including social and communicative deficits, repetitive behaviours, and sensory abnormalities, as well as hyperactivity.

## Introduction

Autism spectrum disorder (ASD) is a common developmental disorder characterised by core symptoms including social and communicative impairments, and stereotyped/repetitive behaviours^1^. In addition to core symptoms, individuals with ASD often have mental health problems, including hyperactivity/inattention (attention deficit hyperactivity disorder; ADHD), aggression, anxiety, cognitive disability, and sleep disorders^1^. Based on the high heritability and complexity of ASD, considerable effort has been put into the identification of genetic mutations associated with ASD. Consequently, a large number of ASD-related genes, which mainly encode proteins for synaptic formation, transcriptional regulation, chromatin remodelling, or actin cytoskeletal dynamics, have been reported^2–4^. Among these genes, several are involved in encoding synaptic cell adhesion molecules, and mice with mutations in those genes exhibit autistic-like behaviours and synaptic dysfunction^5,6^. Recently, genetic studies have revealed that genetic mutations in Kin of Irregular Chiasm-like 3 (KIRREL3) are associated with neurodevelopmental disorders, including intellectual disability and ASD in humans^3,7–12^. KIRREL3 is also one of the synaptic cell adhesion molecules.

Kirrel3 is a member of the mammalian Kirrel gene family, which contains another two members, Kirrel1 and Kirrel2. A *Drosophila* homologue of the mammalian Kirrel gene family, *kirre* (also known as *dumbfounded*), is involved in the muscle cell fusion process as a chemoattractant during embryonic development^13,14^. Previously, we have isolated mouse Kirrel3 (also initially referred to as mKirre) as a mammalian homologue of *kirre*^15^, and have demonstrated that it is highly expressed in the developing and adult nervous systems in mice^16,17^. In addition, some other investigators have reported that Kirrel3 is involved in various processes of neural circuit formation, including neuronal migration, axonal fasciculation, and synapse formation^18–21^. Recently, Choi *et al*.^22^ reported that adult Kirrel3-knockout (Kirrel3^−/−^) mice exhibit moderate hyperactivity and impaired novel object recognition memory in spite of normal synaptic function in the hippocampus and dentate gyrus. However, it remains unclear whether disruption of the Kirrel3 gene is associated with autistic-like behaviours, such as social and communicative impairments, stereotyped/repetitive behaviours, and sensory abnormalities. In the present study, we generated global Kirrel3^−/−^ mice and investigated the behavioural phenotypes relevant to ASD to provide molecular insights into the relationship between Kirrel3 deficiency and ASD. Behavioural battery tests using Kirrel3^−/−^ mice revealed several behavioural phenotypes relevant to ASD, including social and communicative deficits, repetitive behaviours, and sensory abnormalities, as well as hyperactivity.

## Results

### Generation of Kirrel3^−/−^ mice

**Figure 1:**
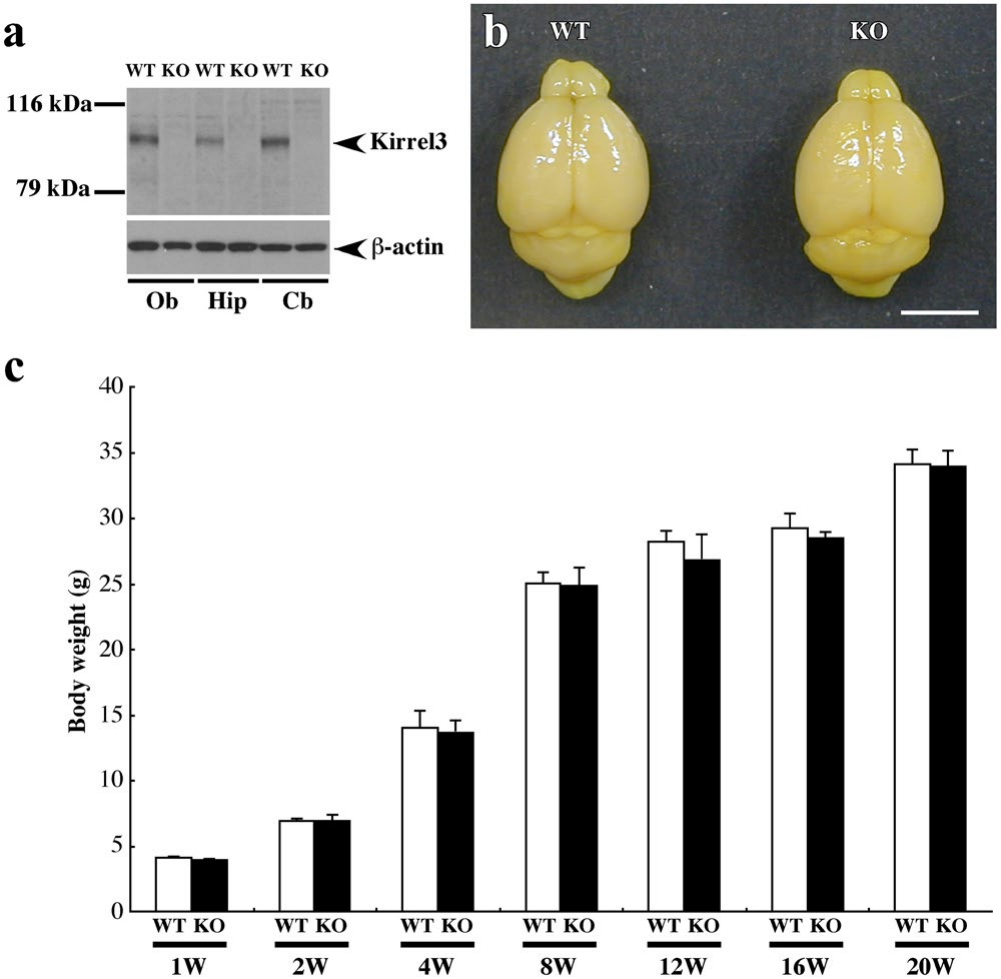
Similar gross morphology of the brain and body weights between wild-type and Kirrel3^−/−^ mice. (**a**) Western blots of olfactory, hippocampal, and cerebellar homogenates from wild-type (WT) and Kirrel3^−/−^ (KO) mice probed with a Kirrel3 antibody. A single band (100 kDa: arrow) was detected in the olfactory bulb (Ob), hippocampus (Hip), and cerebellum (Cb) in wild-type mice, but not in Kirrel3^−/−^ mice. β-actin was used as the loading control. Full-length blots are shown in Supplementary Fig. S2a. (**b**) Gross morphology of adult brain from wild-type (WT) and Kirrel3^−/−^ (KO) mice. There were no differences between genotypes. Scale bar, 5 mm. (**c**) Body weights of wild-type (WT) and Kirrel3^−/−^ (KO) mice throughout development. There were no differences between genotypes (n = 5–14, at each age; *p* > 0.05 at all ages, one-way ANOVA). Error bars indicate SEM.

We first generated Kirrel3^−/−^ mice to determine whether Kirrel3 deficiency causes abnormal behaviours, including autistic-like behaviours (Supplementary Fig. S1a). The correct targeting and integration was confirmed by genotyping using polymerase chain reaction (PCR) (Supplementary Fig. S1b). To examine the lack of Kirrel3 protein in the brain of Kirrel3^−/−^ mice, we performed western blot analysis using an anti-Kirrel3 antibody. Although the expression of Kirrel3 protein was observed in the olfactory bulb, hippocampus, and cerebellum of wild-type mice, no expression of Kirrel3 protein was detected in those of Kirrel3^−/−^ mice (Fig. 1a). There were no obvious differences in the gross appearance or size of the brain between wild-type and Kirrel3^−/−^ mice (Fig. 1b). Histological examination with haematoxylin-eosin staining showed no significant differences in the morphology of the olfactory bulb (Supplementary Fig. S1c,d), cerebral cortex (Supplementary Fig. S1e,f), hippocampus (Supplementary Fig. S1e,f), or cerebellum (Supplementary Fig. S1g,h). The body weights did not differ significantly between wild-type and Kirrel3^−/−^ mice until 20 weeks of age (Fig. 1c). Before the detailed behavioural assays, visual acuity was evaluated using visual placing responses, because poor visual acuity influences the results of most behavioural tests. When suspended by the tail and lowered toward the table, both wild-type and Kirrel3^−/−^ mice raised their heads and reached out their forelimbs for the surface of the table, indicating no difference in visual acuity between genotypes. Therefore, we used wild-type and Kirrel3^−/−^ mice for the following behavioural assays.

### Deficits of social behaviours in Kirrel3^−/−^ mice

**Figure 2:**
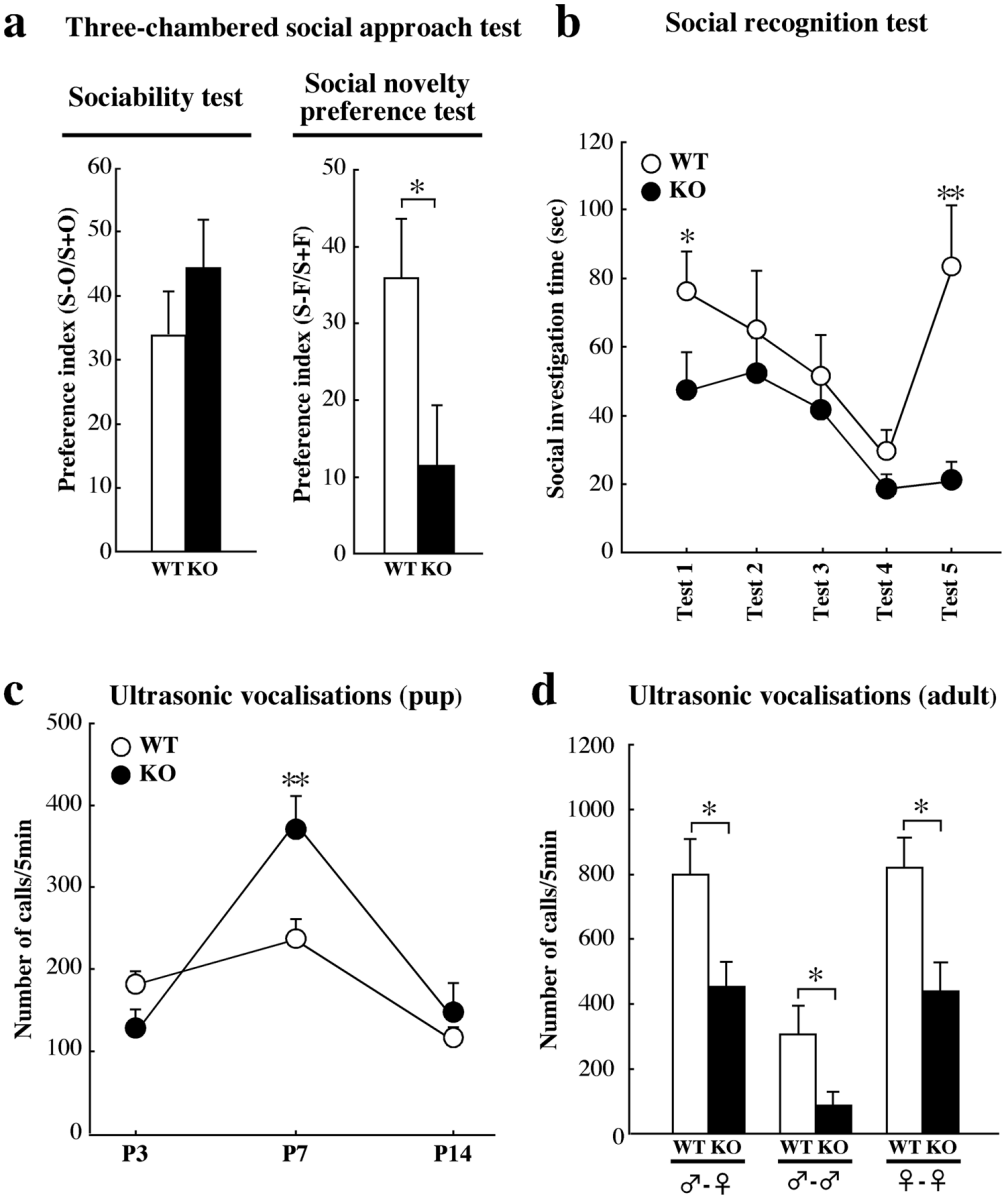
Abnormalities in social recognition and communication behaviours in Kirrel3^−/−^ mice. (**a**) The three-chambered social approach test. The preference indexes in the sociability (left panel) and the social novelty preference tests (right panel) were calculated as described in the Methods. The preference index in the social novelty preference test was significantly lower in Kirrel3^−/−^ (KO) mice compared with that of wild-type (WT) mice (*p < 0.05; one-way ANOVA with Tukey’s *post hoc* test). (**b**) The social recognition test. Compared with wild-type (WT) mice, Kirrel3^−/−^ (KO) mice showed significantly less time investigating a novel unfamiliar mouse at both test 1 (wild-type n = 13, Kirrel3^−/−^ n = 12, *p < 0.05; one-way ANOVA with Tukey’s *post hoc* test) and test 5 (**p < 0.01). (**c**) USV recording during the postnatal stages. At P7, the number of USVs emitted by Kirrel3^−/−^ (KO) pups was significantly increased compared with wild-type (WT) pups (wild-type n = 16, Kirrel3^−/−^ n = 15, **p < 0.01; one-way ANOVA with Tukey’s *post hoc* test). (**d**) USV recording in adulthood. The number of USVs during male-female (wild-type n = 10, Kirrel3^−/−^ n = 10, *p < 0.05; one-way ANOVA with Tukey’s *post hoc* test) or same-sex interactions were significantly decreased in the Kirrel3^−/−^ (KO) mice compared with wild-type (WT) mice (male-male; wild-type n = 10, Kirrel3^−/−^ n = 12, *p < 0.05, female-female; wild-type n = 11, Kirrel3^−/−^ n = 13, *p < 0.05). Error bars indicate SEM.

To examine social behaviours, including the sociability and social novelty preference, in Kirrel3^−/−^ mice, we first performed the three-chambered social approach test^23,24^. The results revealed no significant differences in the distances travelled in the acclimation, sociability, and social novelty preference phases between wild-type and Kirrel3^−/−^ mice (Supplementary Fig. S3a). In the sociability test, a novel unfamiliar (stranger) mouse was placed within a wire cage, which permitted visual, olfactory, auditory, and some tactile contact without fighting^25^, in one side of the three-chamber apparatus. An empty wire cage was placed in the other side of three-chamber as a non-social and inanimate object. Both wild-type and Kirrel3^−/−^ mice spent significantly more time (wild-type n = 11 **p < 0.01, Kirrel3^−/−^ n = 11, ***p < 0.001; one-way ANOVA with Tukey’s *post hoc* test) around the cage containing a stranger mouse (wild-type; 180.6 ± 19.8 sec, Kirrel3^−/−^; 193.4 ± 25.7 sec) than the empty cage (wild-type; 90.1 ± 13.3 sec, Kirrel3^−/−^; 63.1 ± 7.1 sec), and there was no significant difference in the preference index between genotypes (Fig. 2a). In the social novelty preference test, the first stranger mouse used in the sociability test remained within the same cage and served as a familiar mouse. A novel unfamiliar mouse was introduced as a novel stranger mouse within another wire cage in the other side of the three-chamber apparatus. Although wild-type mice spent significantly more time around the cage containing a novel stranger mouse (171.5 ± 16.5 sec) and showed a significant preference for the novel stranger mouse compared with the familiar mouse (80.6 ± 13.1 sec, ***p < 0.001; one-way ANOVA with Tukey’s *post hoc* test), Kirrel3^−/−^ mice did not show any significant preferences (novel stranger mouse; 152.9 ± 18.5 sec, familiar mouse; 117.8 ± 13.1 sec, *p* = 0.14; one-way ANOVA). The preference index in Kirrel3^−/−^ mice was significantly lower than that of wild-type mice (Fig. 2a).

Next, we performed the social recognition test using the habituation/dishabituation paradigm to further investigate social recognition memory^26,27^. In test 1, Kirrel3^−/−^ mice spent significantly less time sniffing towards a novel mouse compared with wild-type mice (Fig. 2b), suggesting that Kirrel3^−/−^ mice exhibited a reduction of social investigation in the home cage. When the same stimulus mouse was presented repeatedly, social investigation time decreased across four presentations (test 1–test 4) in both wild-type and Kirrel3^−/−^ mice, suggesting that habituation was normal. Upon introducing a novel stimulus mouse at test 5, wild-type mice showed an increase in social investigation time toward a novel mouse compared with that at test 4. In contrast, Kirrel3^−/−^ mice failed to show an increase in social investigation time in test 5 compared with test 4 (Fig. 2b). These results suggest that Kirrel3^−/−^ mice exhibited deficits in social investigation and social recognition memory.

### Abnormalities in social communicative behaviours in Kirrel3^−/−^ mice

When separated from their mother during the postnatal periods, mouse pups commonly exhibit ultrasonic calls. These calls represent an infant-mother social communicative behaviour, and are frequently used to evaluate mouse models of ASD^23^. To evaluate communicative behaviour during postnatal stages, we conducted separation-induced ultrasonic vocalisation (USV) recording in Kirrel3^−/−^ pups. At postnatal day (P) 7, the number of ultrasonic calls emitted by Kirrel3^−/−^ pups significantly increased compared with that by wild-type pups (Fig. 2c).

To examine vocal communication in adult mice, we measured the number of ultrasonic calls during free interactions of a tested male mouse with an oestrous C57BL/6J female mouse. During the male-female courtship interaction, male mice emit USVs, which attract female mice toward the vocalising male mice and reduce female aggressive behaviours towards male mice^28^. The number of ultrasonic calls emitted by adult male Kirrel3^−/−^ mice to oestrous female mice was significantly lower than the number emitted by wild-type mice (Fig. 2d). Next, we examined the number of ultrasonic calls during same-sex (male-male or female-female) interactions in the resident-intruder paradigm. In this paradigm, after an intruder mouse is introduced into a resident home cage, the resident mouse typically emits a great number of USVs to establish a social dominance hierarchy^29^ or social recognition^30^. In both male and female pairs (Fig. 2d), the number of ultrasonic calls emitted by Kirrel3^−/−^ mice was significantly lower than the number emitted by wild-type mice.

### Hyperactivity with thigmotaxis, increased rearing behaviours, and enhanced motor learning in Kirrel3^−/−^ mice

**Figure 3:**
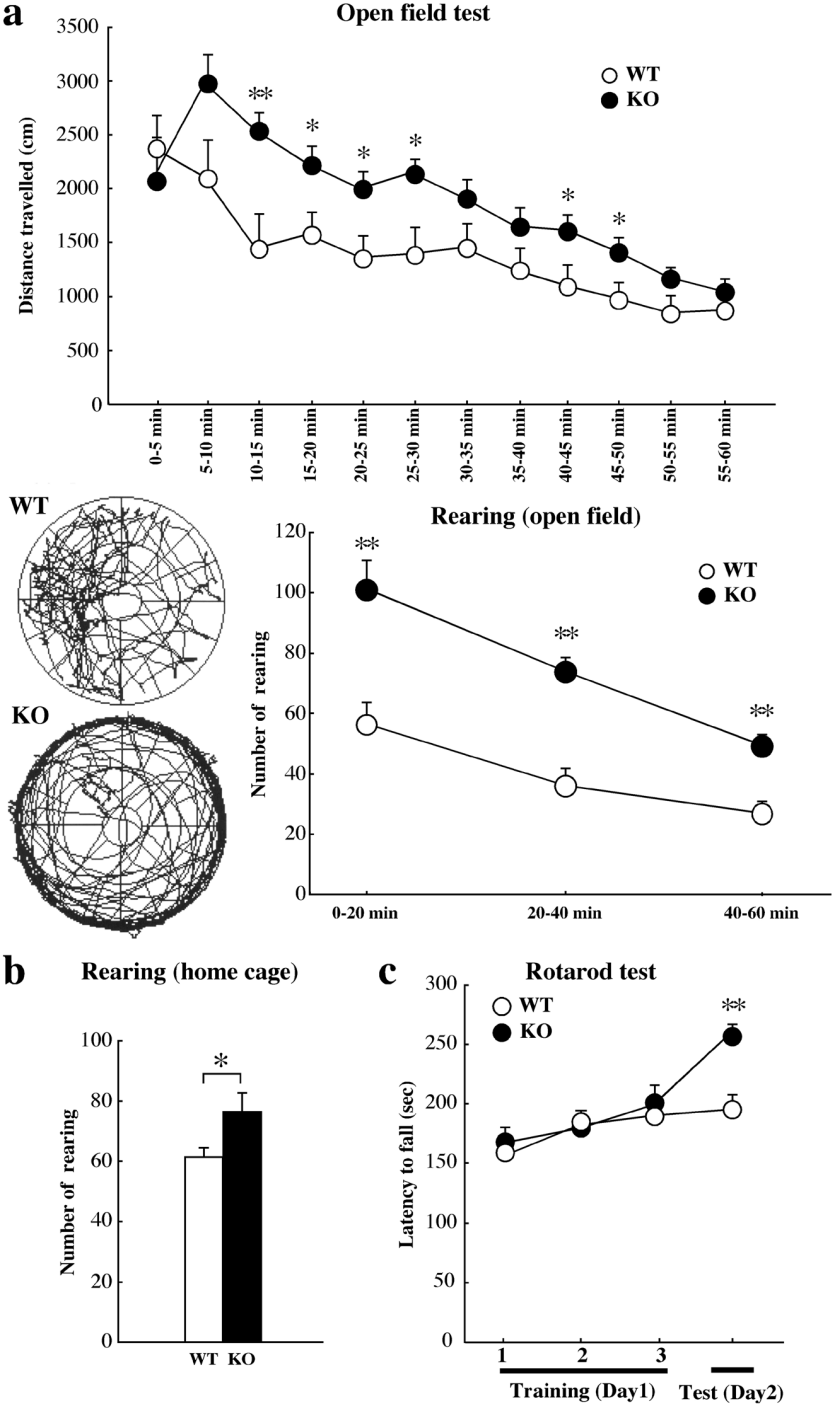
Hyperactivity with thigmotaxis, increased rearing behaviours, and enhanced motor learning in Kirrel3^−/−^ mice. (**a**) The open field test. The distance travelled during the 1 h was significantly higher in Kirrel3^−/−^ (KO) mice than wild-type (WT) mice (upper panel, wild-type n = 12, Kirrel3^−/−^ n = 12, *p* = 0.02; two-way repeated measures ANOVA with Bonferroni-Dunn *post hoc* test, **p < 0.01, *p < 0.05; one-way ANOVA with Tukey’s *post hoc* test at the same sessions). The lower left panel shows representative tracks in the open field test. In the open field, Kirrel3^−/−^ (KO) mice showed thigmotaxis (more exploration at the boundary of the field, and less in the centre). The number of rearing bouts was measured for 1 h (lower right panel). There were significant differences between wild-type (WT) and Kirrel3^−/−^ (KO) mice (*p* = 0.0001; two-way repeated measures ANOVA with Bonferroni-Dunn *post hoc* test, **p < 0.01; one-way ANOVA with Tukey’s *post hoc* test at the same sessions). (**b**) Rearing behaviours in the home cage. The number of rearing bouts was measured for 10 min. There were significant differences between wild-type (WT) and Kirrel3^−/−^ (KO) mice (wild-type n = 8, Kirrel3^−/−^ n = 8, *p < 0.05; one-way ANOVA with Tukey’s *post hoc* test). (**c**) The rotarod test. The latency to fall from the rotating drum was significantly longer in Kirrel3^−/−^ (KO) mice than that of wild-type (WT) mice in the test session (wild-type n = 32, Kirrel3^−/−^ n = 30, **p < 0.01; one-way ANOVA with Tukey’s *post hoc* test). Error bars indicate SEM.

To evaluate spontaneous motor activity, exploratory behaviours, and emotional responses in a novel environment, we performed the open field test. During the 1-hour (h) test session, both distance travelled and rearing behaviours were increased in Kirrel3^−/−^ mice compared with wild-type mice (Fig. 3a). In addition, total distance travelled was also significantly increased in Kirrel3^−/−^ mice (wild-type; 16779.5 ± 1588.7 cm, Kirrel3^−/−^; 23159.3 ± 1919.1 cm, *p < 0.05; one-way ANOVA with Tukey’s *post hoc* test). In a novel, brightly lit arena, mice prefer the periphery to the central areas and tend to run or walk along the wall, a behaviour called thigmotaxis^31^. The percentage of mice exhibiting thigmotaxis was significantly increased (*p < 0.01; chi-square test) in Kirrel3^−/−^ mice (41.1%) compared with wild-type mice (11.1%). In the 24-h home cage activity test, Kirrel3^−/−^ mice exhibited significantly greater locomotor activity during the light phase compared with wild-type mice (Supplementary Fig. S3b). In contrast, although locomotor activity during the dark phase was not significantly different between groups, there was a trend towards reduced activity in Kirrel3^−/−^ mice compared with wild-type mice (Supplementary Fig. S3b). To evaluate repetitive motor behaviours, we analysed grooming, digging, and rearing behaviours in the home cage. Rearing behaviours were significantly increased in Kirrel3^−/−^ mice compared with wild-type mice (Fig. 3b), whereas there were no significant differences in grooming (Supplementary Fig. S3c) and digging (Supplementary Fig. S3d) behaviours between wild-type and Kirrel3^−/−^ mice.

We investigated motor coordination and learning using the rotarod test. There were no significant differences between wild-type and Kirrel3^−/−^ mice in the latency to fall from the rotating drum during the training trials (Fig. 3c). In the test trial, however, the latency was significantly longer in Kirrel3^−/−^ mice (Fig. 3c).

### Normal anxiety-related behaviours, but reduced aggressive behaviours in Kirrel3^−/−^ mice

**Figure 4:**
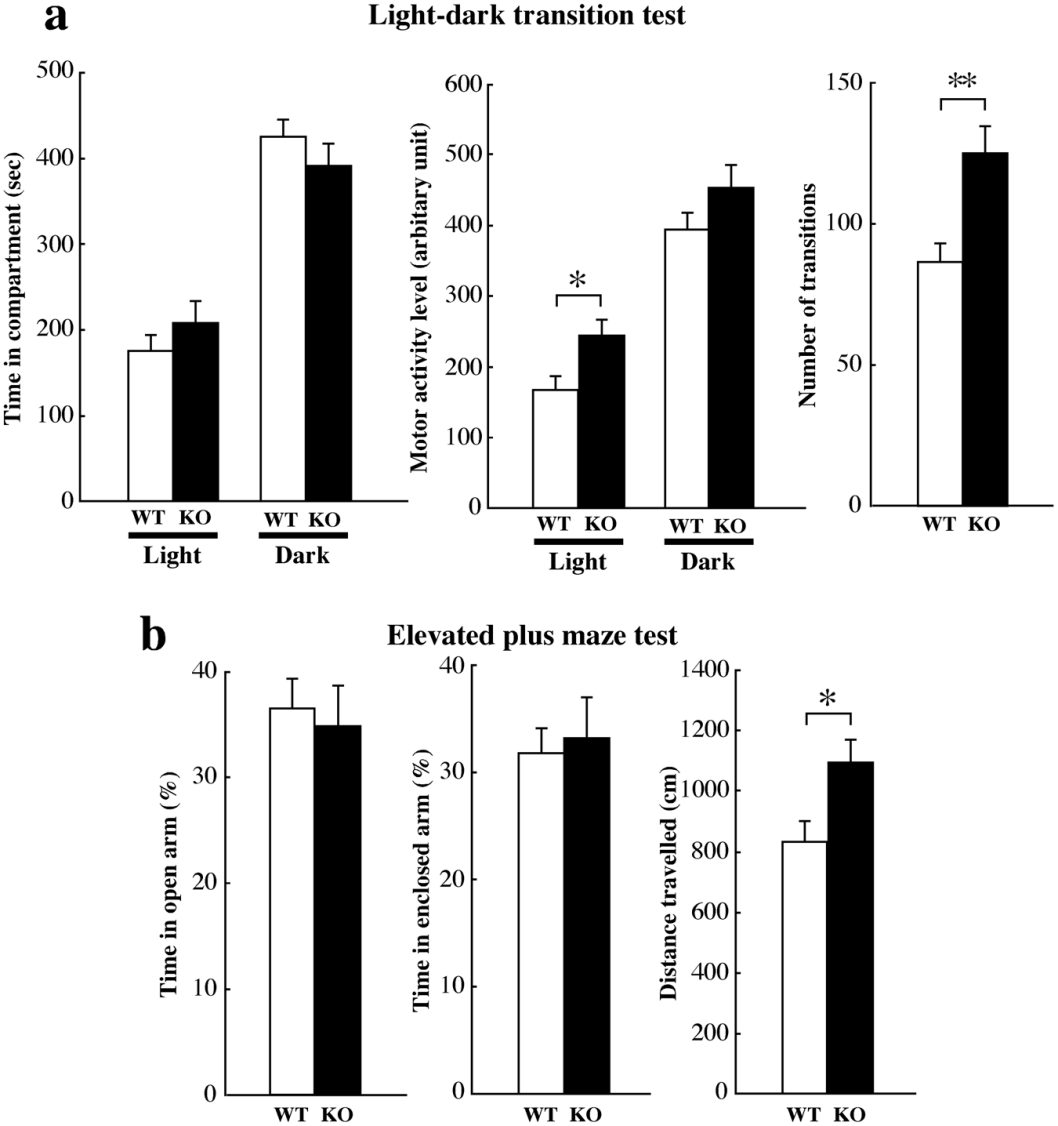
Hyperactivity with normal levels of anxiety in Kirrel3^−/−^ mice. (**a**) The light-dark transition test. There were no significant differences in the time spent in light or dark compartments between wild-type (WT) and Kirrel3^−/−^ (KO) mice (left panel, wild-type n = 13, Kirrel3^−/−^ n = 14, *p* = 0.31; light compartment, *p* = 0.31; dark compartment; one-way ANOVA). Motor activity was significantly increased in the light compartment (middle panel, *p < 0.05; one-way ANOVA with Tukey’s *post hoc* test), but not in the dark compartment (*p* = 0.15). The number of transitions was significantly increased in the light-dark transition test (right panel, **p < 0.01; one-way ANOVA with Tukey’s *post hoc* test). (**b**) The elevated plus maze test. There were no significant differences in the time spent in open (left panel, wild-type n = 13, Kirrel3^−/−^ n = 14, *p* = 0.73; one-way ANOVA) and enclosed arms (middle panel, *p* = 0.75; one-way ANOVA) between wild-type (WT) and Kirrel3^−/−^ (KO) mice. The distance was significantly increased in the elevated plus maze test (right panel; *p < 0.05; one-way ANOVA with Tukey’s *post hoc* test). Error bars indicate SEM.

To investigate anxiety-related behaviours, we performed the light-dark transition test and the elevated plus maze test. In the light-dark transition test, we observed no significant differences in the time spent in the light or dark compartments between wild-type and Kirrel3^−/−^ mice (Fig. 4a). However, Kirrel3^−/−^ mice showed significantly increased motor activity in the light compartment compared with wild-type mice (Fig. 4a), whereas no significant differences were observed in the dark compartment (Fig. 4a). In addition, Kirrel3^−/−^ mice also exhibited significantly more transitions between the light and dark compartments than wild-type mice (Fig. 4a). In the elevated plus maze test, there were no significant differences in the time spent in the open or enclosed arms between wild-type and Kirrel3^−/−^ mice (Fig. 4b). We found that Kirrel3^−/−^ mice exhibited significantly greater locomotor activity than wild-type mice in the elevated plus maze test (Fig. 4b). These results suggest that Kirrel3^−/−^ mice displayed enhanced motor activity without abnormal anxiety-related behaviours.

To evaluate male-male aggression, we performed the resident-intruder test. Consistent with a previous report^20^, Kirrel3^−/−^ mice exhibited lower levels of aggressive behaviour towards C57BL/6J intruder mice (25%) compared with wild-type resident mice (70%, Supplementary Fig. S3e). The attack duration was significantly reduced in Kirrel3^−/−^ mice compared with those in wild-type mice (Supplementary Fig. S3e).

### Abnormal sensory-induced responses to acoustic, olfactory, and pain stimuli in Kirrel3^−/−^ mice

**Figure 5:**
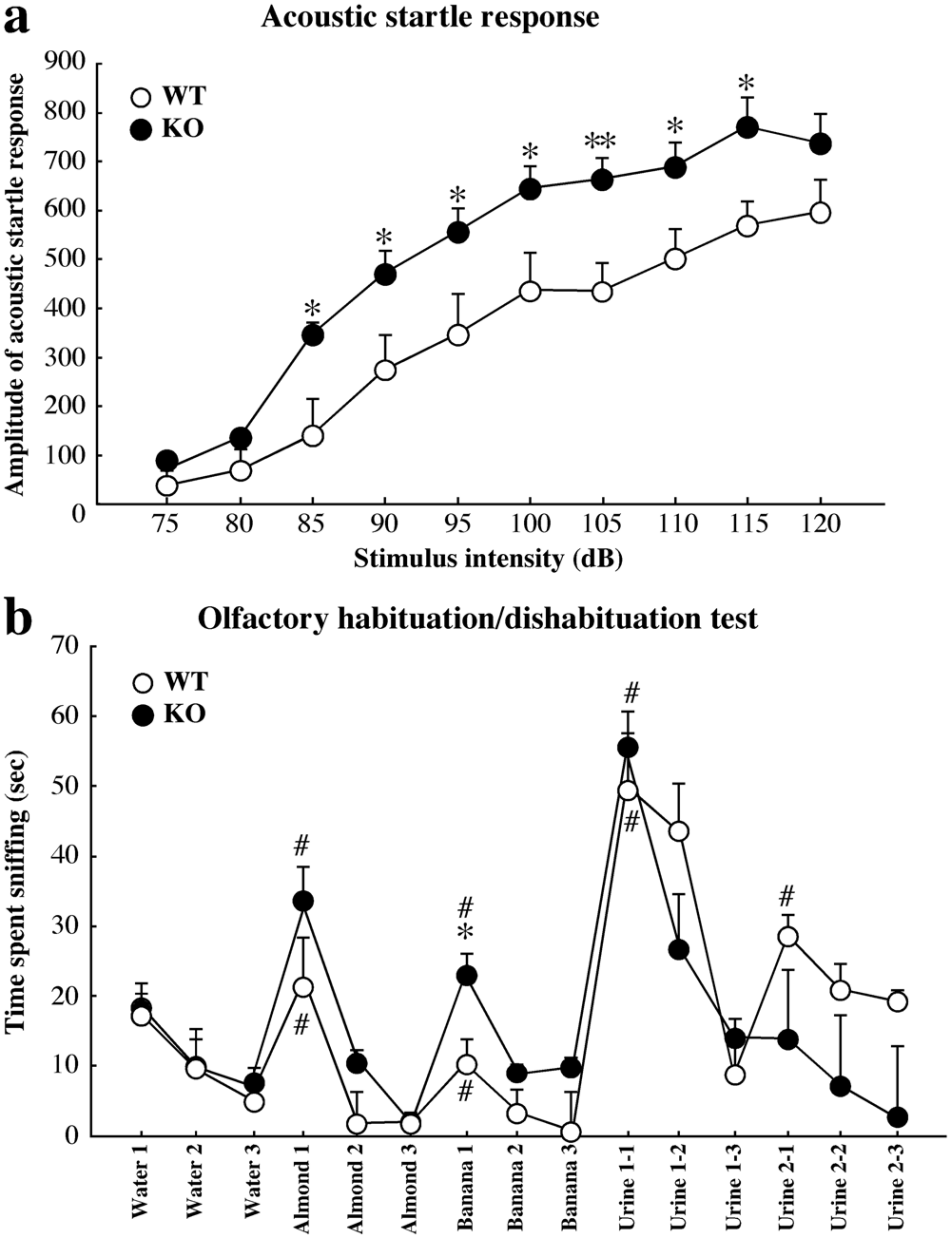
Abnormal sensory-induced responses to acoustic stimuli and social odour discrimination deficits in Kirrel3^−/−^ mice. (**a**) Acoustic startle responses. Stimulus intensity-response magnitude curves in the ASR. The average magnitude of the ASR to acoustic stimuli was significantly greater in Kirrel3^−/−^ (KO) mice than in wild-type (WT) mice (wild-type n = 15, Kirrel3^−/−^ n = 15, *p* = 0.0068; two-way repeated measures ANOVA with Bonferroni-Dunn *post hoc* test, **p < 0.01, *p < 0.05; one-way ANOVA with Tukey’s *post hoc* test in the same sessions). (**b**) The olfactory habituation/dishabituation test. Wild-type (WT) mice spent significantly higher levels of sniffing first presentation of second social odour (urine 2-1) compared with third presentation of first social odour (urine 1-3) (wild-type n = 13, ^#^*p* < 0.05 for dishabituation; one-way ANOVA with Tukey’s *post hoc* test), whereas Kirrel3^−/−^ (KO) mice showed no dishabituation between first social odour and second social odour (Kirrel3^−/−^ n = 16, *p* = 0.97 for dishabituation; one-way ANOVA). Note that Kirrel3^−/−^ mice spent significantly more time sniffing with banana flavouring compared with wild-type mice (wild-type n = 13, Kirrel3^−/−^ n = 16, *p < 0.05; one-way ANOVA with Tukey’s *post hoc* test). Error bars indicate SEM.

One of the major symptoms of ASD is the presence of sensory abnormalities, including hyper-/hyposensitivity to auditory, visual, and tactile stimuli^32^. To examine auditory sensory deficits in Kirrel3^−/−^ mice, we measured the acoustic startle response (ASR) to acoustic stimuli ranging from 75 to 120 dB. The average magnitude of the ASR was significantly greater in Kirrel3^−/−^ mice between 85 dB and 115 dB (Fig. 5a). In addition, sensorimotor gating was assessed using the prepulse inhibition (PPI) test. We observed no significant differences in PPI of the ASR between wild-type and Kirrel3^−/−^ mice (Supplementary Fig. S4a).

To evaluate olfactory deficits, we performed the buried food-finding test. The test revealed no significant differences in the time required to find buried food between wild-type and Kirrel3^−/−^ mice (Supplementary Fig. S4b). Olfactory perception is also important for social behaviours^33^, including social interaction and social recognition in mice. Using the olfactory habituation/dishabituation test, we further evaluated whether Kirrel3^−/−^ mice were able to detect and discriminate non-social and social odours. Compared with wild-type mice, Kirrel3^−/−^ mice exhibited normal olfactory habituation/dishabituation for non-social odours (water, almond, and banana). The first presentation of a non-social odour elicited moderate sniffing that decreased across three presentations of the same non-social odour (Fig. 5b). The presentation of a distinct non-social odour elicited significantly more sniffing, which also decreased across three presentations of the same odour. A social odour following to the non-social odours also elicited high levels of sniffing that decreased across three presentations of the same social odour in both wild-type and Kirrel3^−/−^ mice (Fig. 5b). When a new odour was introduced, more sniffing was elicited than the third presentation of the previous odour in both genotypes. However, when the second social odour was introduced, wild-type mice showed significantly more sniffing compared with the third presentation of the first social odour, but Kirrel3^−/−^ mice did not (Fig. 5b). These findings indicate that Kirrel3^−/−^ mice had a normal sense of smell, but were deficient in the discrimination and/or memory for social odours.

To investigate pain sensitivity, we measured the foot shock current required to elicit flinching, vocalising, and jumping behaviours. The level of current required to elicit vocalisation was significantly higher in Kirrel3^−/−^ mice compared with wild-type mice (Supplementary Fig. S4c). However, there were no differences in the level of current required to elicit flinching and jumping behaviours between wild-type and Kirrel3^−/−^ mice (Supplementary Fig. S4c).

### Normal spatial or fear learning/memory with behavioural flexibility in Kirrel3^−/−^ mice

We examined spatial learning and memory in Kirrel3^−/−^ mice using the Morris water maze test. In the test, the mouse swims to find a hidden platform in a pool with prominent extramaze cues. In the acquisition task, there were no significant differences in the time spent exploring the platform or in the number of platform location crossings between wild-type and Kirrel3^−/−^ mice (Supplementary Fig. S5a). In the reversal task, the target platform was moved to the opposite area to analyse responses to changes in routine^34^. In the reversal task, there were also no significant differences in the time spent exploring platform and in the number of platform location crossing between wild-type and Kirrel3^−/−^ mice (Supplementary Fig. S5b). These results suggest that Kirrel3^−/−^ mice showed no deficits in spatial learning and memory, or behavioural inflexibility.

To examine short-term fear memory, we performed the passive avoidance test. There were no significant differences in the latency to enter the dark compartment 24 h after foot shock between wild-type and Kirrel3^−/−^ mice (Supplementary Fig. S5c), indicating that Kirrel3^−/−^ mice exhibited no deficits of short-term fear memory.

## Discussion

In the present study, Kirrel3^−/−^ mice showed hyperactivity with normal levels of social interaction, grooming, anxiety-like behaviour, spatial learning and memory, which is consistent with a previous study reported by Choi *et al*.^22^. In addition, we found that Kirrel3^−/−^ mice exhibited impaired social recognition, communication deficits with USVs, enhanced repetitive behaviours, and sensory abnormalities.

One of the core symptoms of ASD is impaired social behaviour and social communication^1^. Several mouse models of ASD exhibit the absence of a preference for a mouse over an object in the three-chambered social approach test, which is used as an indicator of impaired social behaviour^23,34–39^. However, Kirrel3^−/−^ mice exhibited normal sociability in the present study, consistent with a previous report using another line of Kirrel3^−/−^ mice^22^. Because autistic patients often avoid unfamiliar social partners and show diminished interest in novelty^1^, an impairment of social novelty preference (preference for a novel mouse over a familiar mouse) in the three-chambered social approach test is used as an indicator of lower social recognition in some ASD mouse models^23,38,40^. In the present study, Kirrel3^−/−^ mice showed an impairment of social novelty preference. Some ASD mouse models lacking ASD-related genes also exhibit normal sociability and impaired social novelty^40–43^. As suggested by Carter *et al*.^40^, the lack of sociability is not always required for a diagnosis of ASD and it might be a symptom of individuals with ASD^1^.

Because the impairment of social novelty preference is related to the impaired social recognition memory observed in some patients with ASD^44^ as well as in some ASD mouse models^26,27,45^, we further performed the social recognition memory test using the habituation/dishabituation paradigm. We found that Kirrel3^−/−^ mice exhibited impaired habituation to a novel mouse and a reduction of novel mouse investigation time. These results suggest that Kirrel3^−/−^ mice exhibited impaired social behaviours, including social recognition deficits and/or social recognition memory deficits.

USVs are emitted as a communicative behaviour by mice in different social contexts, including the separation of pups from the mother, and adult social interactions. Recording of USVs has been used routinely to assess behavioural phenotypes in rodent models of ASD^25^, and it has been reported that alterations in USVs are often observed in ASD mouse models during the postnatal and adult periods. In the present study, Kirrel3^−/−^ mice exhibited a reduction in USVs in both male-female and same-sex interactions in adulthood. Interestingly, Kirrel3^−/−^ mice emitted increased USVs at P7 in separation-induced USV recording. These results indicate that Kirrel3^−/−^ mice showed abnormalities in social communicative behaviours not only in adulthood, but also in developmental stages. Social communicative deficits are typically recognised during the early developmental period in ASD patients, persisting into adulthood. The presence of symptoms in early childhood is a key component of the criteria for diagnosis of ASD^1^. In the present study, Kirrel3^−/−^ mice exhibited early onset of social communicative abnormalities, suggesting that Kirrel3^−/−^ mice may be a suitable model for investigating the neural mechanisms of early ASD symptoms.

Another core symptom of ASD is restricted, repetitive patterns of behaviours, interests, or activities^1^. The increases in grooming, digging, and rearing behaviours are often observed as repetitive behaviours in some ASD mouse models, such as BTBR, Tsc1-knockout, neurexin-1α-knockout, and C58/J mice^36,38,46–48^. In the present study, there were no differences in the levels of grooming and digging behaviours between wild-type and Kirrel3^−/−^ mice, consistent with a previous report^22^. However, we found that Kirrel3^−/−^ mice showed increased rearing behaviours in the home cage. In the open field test, Kirrel3^−/−^ mice exhibited rearing behaviours and thigmotaxis more frequently than wild-type mice during 1-h test session. Although increased anxiety may lead to thigmotaxis^49^, Kirrel3^−/−^ mice showed normal levels of anxiety-related behaviours in the elevated plus maze test and the light-dark transition test. Thigmotaxis was also observed in other ASD mouse models, such as Tbx1-heterozygous mice, in which no anxiety behaviours are exhibited^50^. Based on these findings, we could interpret the increased thigmotaxis observed in Kirrel3^−/−^ mice as a form of repetitive behaviour rather than anxiety-like behaviours. In addition, Kirrel3^−/−^ mice showed better performance in the rotarod test, which has been reported in several mouse models of ASD, and can be interpreted as reflecting enhanced formation of a repetitive motor routine^34,35,37,51^. Taken together, it is possible that these enhanced motor behaviours in Kirrel3^−/−^ mice represent an increase in repetitive/stereotyped behaviours, which is one of the core symptoms of ASD.

It has been recently recognised that sensory processing abnormalities, such as hyper-/hyposensitivity to auditory, visual, and tactile stimuli, represent a core symptom of restricted, repetitive patterns of behaviours, interests, or activities^1,32,52^. Hypersensitivity to auditory stimuli is known to be the most common sensory abnormality impairing behavioural adaptation in patients with ASD^52,53^. In the present study, we observed that Kirrel3^−/−^ mice showed hypersensitive responses to acoustic stimuli. Thus, Kirrel3^−/−^ mice showed impaired social recognition, abnormal USVs, repetitive motor behaviours, and auditory hypersensitivity, which are relevant to the core symptoms observed in patients with ASD^1,52^. The current data indicate that the Kirrel3^−/−^ mouse is a valuable model for investigating the neural mechanisms underlying ASD, such as the synaptic dysfunction and the abnormal connectivity of neural circuits.

The association between KIRREL3 gene disruption and ASD was first suggested in a case of Jacobsen syndrome with ASD, reported by Guerin *et al*.^8^. In their study, a 4-year-old girl with significant delays in receptive and expressive language and difficulties in social interaction and eye contact was diagnosed with ASD^8^. Recently, disruption in the KIRREL3 locus (28 copy number variants) was identified in patients with ASD or neurodevelopmental disorders by sequencing chromosomal abnormalities^9^. In that study, a 4.5-year-old girl with a disruption 39.6 kb upstream of the *KIRREL3* gene locus and reduced expression levels of Kirrel3 protein, was diagnosed with neurodevelopmental disorders by deficits in attention, spatial coordination, and speech^9^. As common symptoms of these two patients were communication deficits, Kirrel3 may play an important role in the development of communicative behaviours. Furthermore, whole-genome sequencing in monozygotic twins with ASD and their parents identified *de novo* point mutations in the KIRREL3 gene^10^. Exome sequencing of 343 families with a single child with ASD and at least one affected sibling revealed three *de novo* missense mutations in the KIRREL3 gene^11^. However, the association of these KIRREL3 gene mutations with each symptom of ASD remains to be elucidated.

In the present study, we also found that Kirrel3^−/−^ mice exhibited hyperactivity in novel bright environments across some behavioural tests, including the open field test, the elevated plus maze test, and the light-dark transition test. In contrast, Choi *et al*. reported that Kirrel3^−/−^ mice exhibited moderate hyperactivity in a familiar (home cage) environment, but not in a novel (dark open field) environment^22^. The discrepancy between the current observation and these previous findings may be due to the different conditions of the novel environments used in each study, such as the illumination of the room and the apparatus. In the previous study, the open field test was performed under dark (complete darkness and ~20 lux) illumination using a smaller square open field box, whereas we used a larger circular open-field arena under bright (600 lux) illumination. Because Kirrel3^−/−^ mice also exhibited hyperactivity in the small square light-dark box (17 × 15 × 16 cm) under bright (600 lux) illumination in the dark-light transition test, the different results between the previous study and the present study may be caused by differences in illumination but not the size and shape of the arena. Indeed, we observed that Kirrel3^−/−^ mice did not show hyperactivity under dim-light conditions (120 lux) in the three-chambered social approach test. Furthermore, we found that Kirrel3^−/−^ mice were also hyperactive in both a familiar environment (home cage) during the light phase, but not in the dark phase. Similarly, Kirrel3^−/−^ mice were also more hyperactive in the light box (600 lux), but not in the dark box (50 lux) in the light-dark transition test. These findings indicate that Kirrel3^−/−^ mice may become more hyperactive in bright environments. Because most lines of mice show an aversion to brightly illuminated areas^54^, hyperactivity of Kirrel3^−/−^ mice may reflect a strong escape behaviour from an aversive environment. Alternatively, it is possible that Kirrel3^−/−^ mice have abnormal sensitivity to light stimuli, which is relevant to the symptoms of sensory processing abnormality observed in ASD^1,52,53^.

ASD patients are often comorbid with hyperactivity/inattention, aggression, anxiety, cognitive disability, and sleep disorders^1^. Recently, the existence of hyperactivity/inattention in ASD patients is diagnosed as ASD comorbid with ADHD^1^. When comorbid with ADHD, ASD patients show severe autistic symptoms and poor outcomes after treatment for ASD^55–58^, which is a serious concern in the clinical assessment and treatment of ASD. In the present study, Kirrel3^−/−^ mice exhibited hyperactivity in addition to autistic-like behaviours. Furthermore, Kirrel3^−/−^ mice had an impaired discrimination of small stimulus differences (between novel and familiar objects and between novel and familiar mice), but not large stimulus differences (between objects and mice) (22, present study). Because attention is important for discriminating small stimulus differences^59^, one possible explanation of these discrimination deficits is that Kirrel3^−/−^ mice may suffer from inattention. Recently, it has been reported that Brinp1-knockout mice and cyclin-dependent kinase-like 5-knockout mice are mouse models of ASD comorbid with ADHD^60,61^. One of the different phenotypes between these mice and Kirrel3^−/−^ mice is that Kirrel3^−/−^ mice do not show impaired learning. Previous studies reported that ASD patients with normal or high intelligence quotients are often comorbid with ADHD^62,63^. Although further characterisation of Kirrel3^−/−^ mice for ADHD-like behaviours, such as inattention and impulsivity, will be required, Kirrel3^−/−^ mice may be represented a mouse model of ASD comorbid with ADHD and could provide molecular and pathophysiological information, and insight into new treatments for ASD comorbid with ADHD.

In conclusion, Kirrel3^−/−^ mice exhibited autistic-like behaviours, including social and communicative deficits, repetitive motor behaviours, and auditory hypersensitivity, as well as hyperactivity. In addition, Kirrel3^−/−^ mice also showed normal anxiety-related behaviours and spatial or fear learning/memory.

## Methods

### Mice

Heterozygous Kirrel3-knockout (Kirrel3^+/−^) mice were generated on a C57BL/6 background by Ozgene Pty Ltd (Bentley DC, Australia) as described in the Supplementary Methods. Behavioural tests were performed with adult male wild-type and Kirrel3^−/−^ mice (11–32 weeks old through the entire experiment), unless otherwise indicated. In the analyses for grooming and rearing behaviours, young (5–10 weeks old) male mice were also used. In USV recording, both male and female mice (16–20 weeks old) and pups (P3, P7, P14) were used. All behavioural tests were performed blind to genotypes with age-matched littermate pairs in accordance with standard procedures. Mice were maintained on a 12:12 h light/dark cycle with lights on from 8 a.m. to 8 p.m., and had access to food and water *ad libitum* All behavioural tests were performed during the light phase except for USV recording in adult mice. Wild-type and Kirrel3^−/−^ mice were housed together in the same cage (3–4 mice per cage) in the animal colony room. They were then individually housed for at least 3 days before the first behavioural test.

At all times, the experiments were carried out in accordance with the Guidelines for Animal Experiments of Wakayama Medical University, Japanese Government Notification on Feeding and Safekeeping of Animals (No. 6), and the National Institute of Health Guide for the Care and Use of Laboratory Animals (NIH Publications No. 80-23 revised 1978). All efforts were made to minimise the number of animals used and their suffering. All experimental protocols were approved by the Animal Research Committee of Wakayama Medical University.

### Three-chambered social approach test

To investigate sociability (tendency to social contact with a stranger mouse versus an inanimate object) and social novelty preference (tendency to social contact with a novel stranger versus a familiar mouse), the three-chambered social approach test was performed, as described previously^24,39^. The three-chamber apparatus (O’Hara & Co., Tokyo, Japan) was a non-transparent white plastic box with two transparent acrylic partitions with a rectangular opening (5 × 3 cm). The acrylic partitions divided the box into three chambers (left, centre, and right; 20 × 40 × 22 cm each), which were illuminated at 120 lux. Each side chamber contained a cylindrical wire cage (9 cm diameter × 10.5 cm height) in the corner to hold a stimulus mouse. The wire cage consisted of vertical bars, allowing minimal contact between mice, and preventing fighting. A white cup was placed on the top of the wire cage to prevent the test mouse from climbing to the top of the cage. All stranger mice were age-matched male C57BL/6J mice, which were acclimated to the wire cages for 10 min before beginning the test. The test consisted of three phases: acclimation, sociability, and social novelty preference. During the acclimation phase, a test mouse was placed in the centre chamber and allowed to freely investigate and habituate to all three chambers and wire cages for 10 min. A stranger mouse was then placed in one of the wire cages. During the second phase, the test mouse was placed in the centre chamber and allowed to freely investigate all three chambers for 10 min (sociability test). Subsequently, a novel stranger mouse was placed in the wire cage that had been empty during the sociability test. During the third phase, the test mouse was placed in the centre chamber and allowed to freely investigate all three chambers for 10 min (social novelty preference test). Movement of the test mouse was recorded with an infrared video camera and movement tracks were analysed with Image J CSI software that modified the Image J program (O’Hara & Co.). The time spent in close interaction with each wire cage was converted into a preference index. The preference index in the sociability test was calculated as follows: ([time spent exploring the stranger mouse] − [time spent exploring the object])/[total time spent exploring both targets] × 100. The preference index in the social novelty preference test was calculated as follows: ([time spent exploring the stranger mouse] − [time spent exploring the familiar mouse])/[total time spent exploring both targets] × 100.

### Social recognition test

The social recognition test was performed as described previously^26,27^ with some modifications. Each test mouse was transferred into individual housing for 3 days before testing. A stimulus mouse contained in a cylinder with holes was introduced into the home cage of each test mouse for a 5-min confrontation. Tests were repeated 5 times (tests 1–5) at intervals of 15 min. All stimulus mice were age-matched C57BL/6J male mice. In tests 1–4, the same stimulus mouse was introduced, and a novel stimulus mouse was introduced in test 5. The behaviours were video-recorded and the time spent sniffing the stimulus mouse was measured with a stopwatch.

### USV recording

In a soundproof chamber (O’Hara & Co.), ultrasonic sounds were detected by a condenser ultrasound microphone (UltraSoundGate CM16/CMPA, Avisoft Bioacoustics, Berlin, Germany) connected to an A/D converter (UltraSoundGate 116Hb, Avisoft Bioacoustics). Acoustic signals were recorded with an Avisoft SASLab Pro Recorder (Avisoft Bioacoustics) and transmitted to a sound analysis system (Avisoft SASLab Pro, Avisoft Bioacoustics).

To evaluate communication-related behaviour during postnatal stages, we monitored maternal separation-induced USVs as described previously^34^ with some modifications. Each pup was isolated from their mother and placed in a small cage containing fresh bedding. The number of USVs for 5 min was counted manually.

To examine adult vocal communication during different-sex interactions, we monitored courtship USVs as described previously^39^. To induce courtship USVs in male mouse, an oestrous female C57BL/6J mouse was introduced into the male home cage. The number of USVs in a 5-min period was counted manually.

To further examine adult vocal communication during same-sex social interactions, we monitored USVs in the resident-intruder test as described previously^28–30^ with some modifications. An intruder C57BL/6J mouse was introduced to the resident home cage. The number of USVs in a 5-min period was counted manually.

### Open field test

The open field test was performed as described previously^34,39^. Each test mouse was placed in the centre of an open field arena (75 cm diameter × 40 cm height) illuminated at 600 lux and allowed to explore for 1 h. The total distance travelled and the distance travelled in every 5 min was evaluated with a computerised video-tracking system (CompACT VAS system; Muromachi Kikai, Tokyo, Japan). The number of rearing behaviours was counted manually.

### Light-dark transition test

The light-dark transition test was conducted as described previously^34,39^. The light-dark box (Med Associates, St. Albans, VT) was divided into two compartments (17 × 15 × 16 cm each) by a black partition with two rectangular openings (6 × 6 cm each); one compartment was white and brightly illuminated (600 lux), and the other was black and dark (50 lux). Each test mouse was placed into the light compartment and allowed to move freely between the two compartments for 10 min. The time spent in the light or dark compartment, the motor activity levels in the light or dark compartment, and the number of transitions were recorded using MED-PC IV software (Med Associates).

### Elevated plus maze test

The elevated plus maze test was conducted as previously described^34,39^. The elevated plus-maze was elevated to a height of 50 cm above the floor and consisted of two open arms and two enclosed arms (25 × 8 cm each) that extended from a central area (8 × 8 cm). The enclosed arms were surrounded by 20-cm-high walls. Each test mouse was placed in the central area facing one of the open arms and allowed to explore for 5 min. The time spent in open or enclosed arm and the distance travelled were analysed using a computerised video-tracking system (CompACT VAS system).

### ASR and PPI test

A startle reflex measurement system (SR-LAB; San Diego Instruments, San Diego, CA) was used as described previously^34,46^ with some modifications. Each test mouse was placed in a Plexiglas cylinder and acclimated to the experimental conditions. Each test session consisted of a 5-min acclimation period with only background noise (70 dB) followed by startle sounds with 40 millisecond (msec) duration at intensities varying from 75 to 120 dB and repeated 5 times/day for 3 consecutive days. The peak startle amplitude recorded for 200 msec starting with the onset of the startle sound was used as the magnitude of the ASR. For the PPI test, a prepulse sound was presented 20-msec before the 115 dB startle sound, and its intensity was 74 or 78 dB. A percentage of PPI for each trial was calculated as follows: ([ASR on startle sound alone] − [ASR on prepulse plus startle sounds])/[ASR on startle sound alone] × 100. Trials were performed in a pseudorandom order with random intertrial intervals (10–20 sec).

### Statistical analyses

All statistical analysis was conducted using Excel 2011 (Microsoft, Seattle, WA) with the add-on software Statcel 3 (OMS publishing, Saitama, Japan). Data are presented as mean ± standard error of the mean (SEM). Data were compared using one-way analysis of variance (ANOVA) with Tukey’s *post hoc* test, repeated measures ANOVA with Bonferroni-Dunn *post hoc* test, or chi-square (*x*^2^) test. A value of *p* < 0.05 was considered to represent a significant difference for all tests.

## Electronic supplementary material

Supplementary Information
