# Supplementary material for: Abnormal behaviours relevant to neurodevelopmental disorders in Kirrel3-knockout mice
Source: Sci Rep. 2018 Jan 23;8:1408. doi: 10.1038/s41598-018-19844-7 (PMC5780462; doi:10.1038/s41598-018-19844-7)
Supplement: Supplementary file 1 — Supplementary Information [file 41598_2018_19844_MOESM1_ESM.pdf]

Supplementary Information

Abnormal behaviours relevant to neurodevelopmental disorders in Kirrel3-knockout mice

Tomoko Hisaoka[1], Tadasuke Komori[1], Toshio Kitamura[2], Yoshihiro Morikawa[1]*

[1]Department of Anatomy and Neurobiology, Wakayama Medical University, 811-1 Kimiidera, Wakayama 641-8509, Japan.

[2]Division of Cellular Therapy, Advanced Clinical Research Center, The Institute of Medical Science, The University of Tokyo, 4-6-1 Shirokanedai, Minato-ku, Tokyo 108-8639, Japan.

*Correspondence to: Yoshihiro Morikawa, Department of Anatomy and Neurobiology, Wakayama Medical University, 811-1 Kimiidera, Wakayama 641-8509, Japan.

Tel & Fax: +81-73-441-0617

E-mail: yoshim@wakayama-med.ac.jp

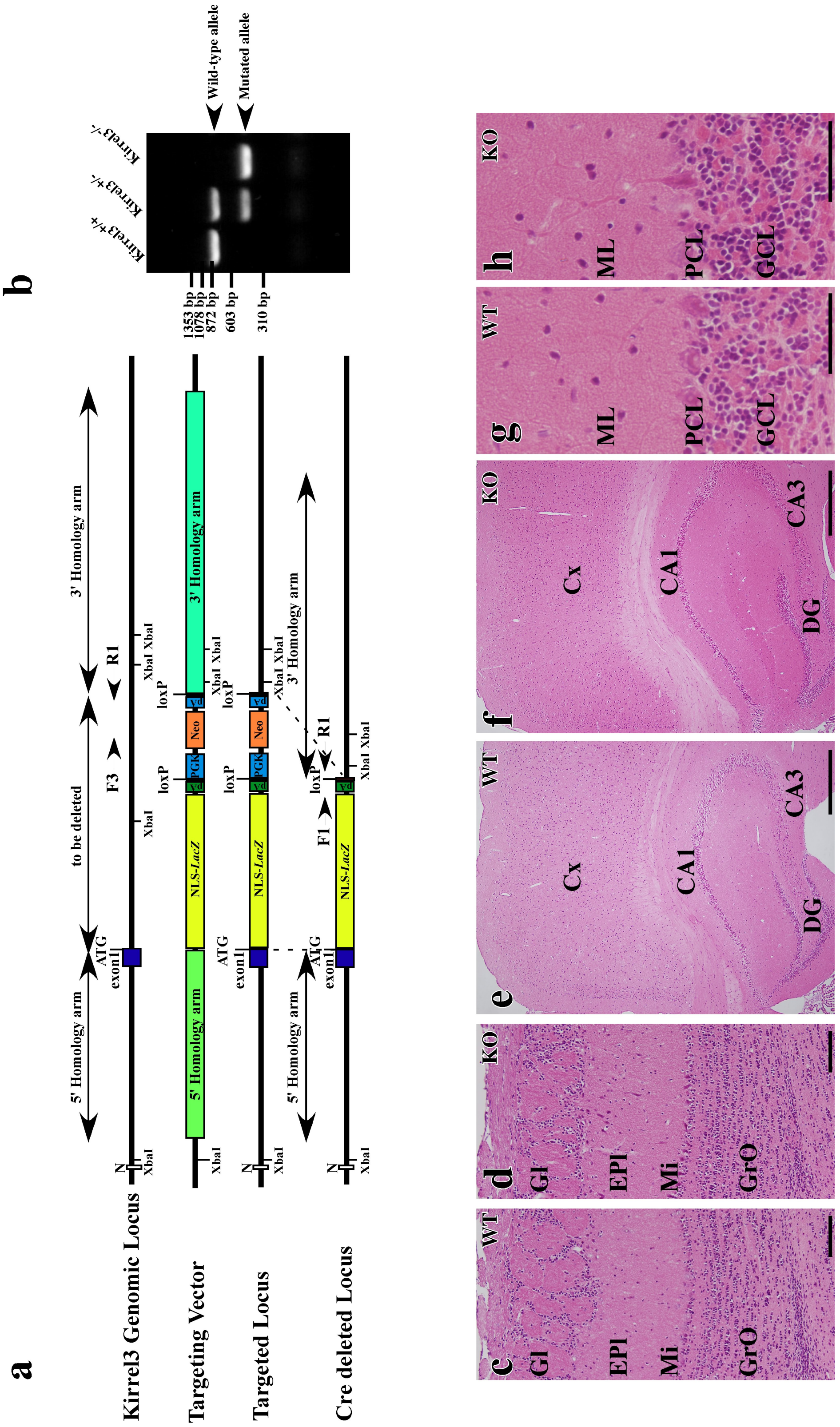
a
b
Kirrel3 Genomic Locus
Targeting Vector
Targeted Locus
Cre deleted Locus
5' Homology arm
to be deleted
3' Homology arm
5' Homology arm
3' Homology arm
NLS-LacZ
PGK
Neo
loxP
ATG
exon1
XbaI
F3
R1
F1
1353 bp
1078 bp
872 bp
603 bp
310 bp
Kirrel3+/+
Kirrel3+/-
Kirrel3-/-
Wild-type allele
Mutated allele
c
d
e
f
g
h
WT
KO
Gl
EPl
Mi
GrO
Cx
CA1
CA3
DG
ML
PCL
GCL

Supplementary Figure S1. Generation of the Kirrel3-knockout/lacZ knockin mice. (a) The wild-type genomic organisation and targeting construct are shown. The targeting construct was designed to replace the Kirrel3 coding region with NLS-LacZ. Downstream of the reporter gene was the selection gene (PGK-Neo) bracketed by loxP recombination signals, which was later deleted by mating with mice expressing Cre recombinase. (b) PCR genotyping showing proper integration of the targeting construct. The positions of bands to detect the wild-type and the mutated alleles are marked by arrowheads. A full-length gel image is shown in Supplementary Fig. S2b. (c-h) Histology of adult olfactory bulb (c, d), cerebral cortex and hippocampus (e, f), and cerebellum (g, h) in wild-type (WT; c, e, g) and Kirrel3$^{-/-}$ (KO; d, f, h) mice by haematoxylin-eosin staining. CA1, field CA1 hippocampus; CA3, field CA3 hippocampus; Cb, cerebellum; Cx, cerebral cortex; DG, dentate gyrus; EPl, external plexiform layer of the olfactory bulb; GCL, granule cell layer; Gl, glomerular layer of the olfactory bulb; GrO, granule cell layer of the olfactory bulb; Hip, hippocampus; Mi, mitral cell layer; ML, molecular layer; Ob, olfactory bulb; PCL, Purkinje cell layer. Scale bars: c-f, 100 μm; g, h, 50 μm.

a

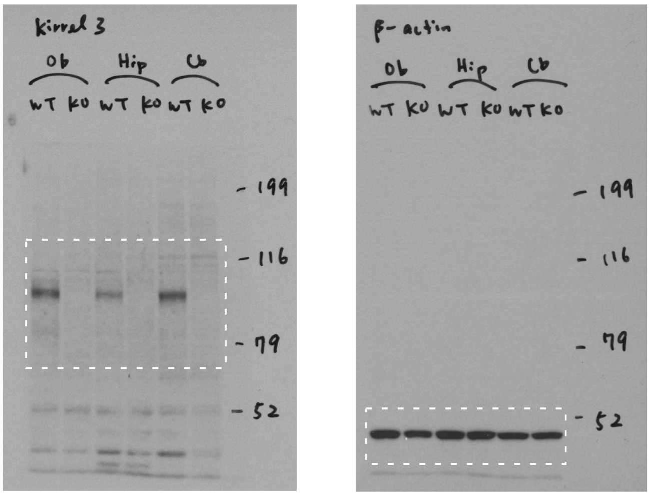

b

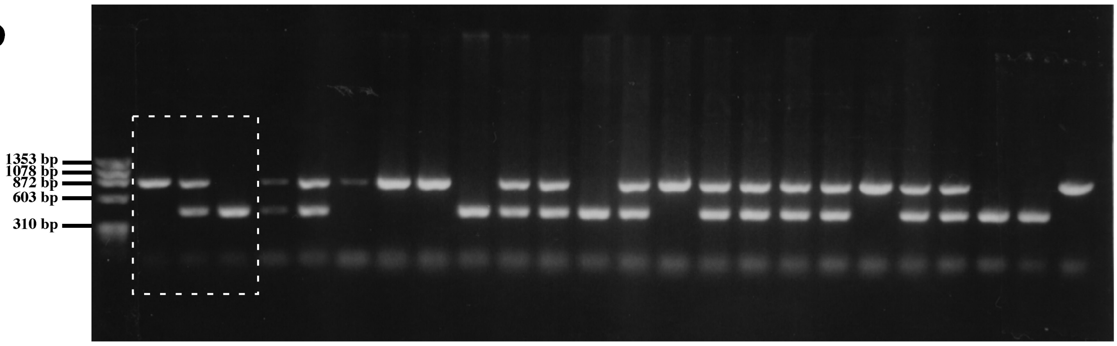

Supplementary Figure S2. Full-length gel and blots shown in Figure 1 and Supplementary Figure S1. (a) Full-length blots presented in Figure 1a are shown. The cropped regions shown in Figure 1a are outlined by white dashed line in each panel (left panel; kirrel3, right panel; β-actin). (b) A full-length gel image presented in Supplementary Figure S1b is shown. The cropped region shown in Supplementary Figure S1b is outlined by a white dashed line.

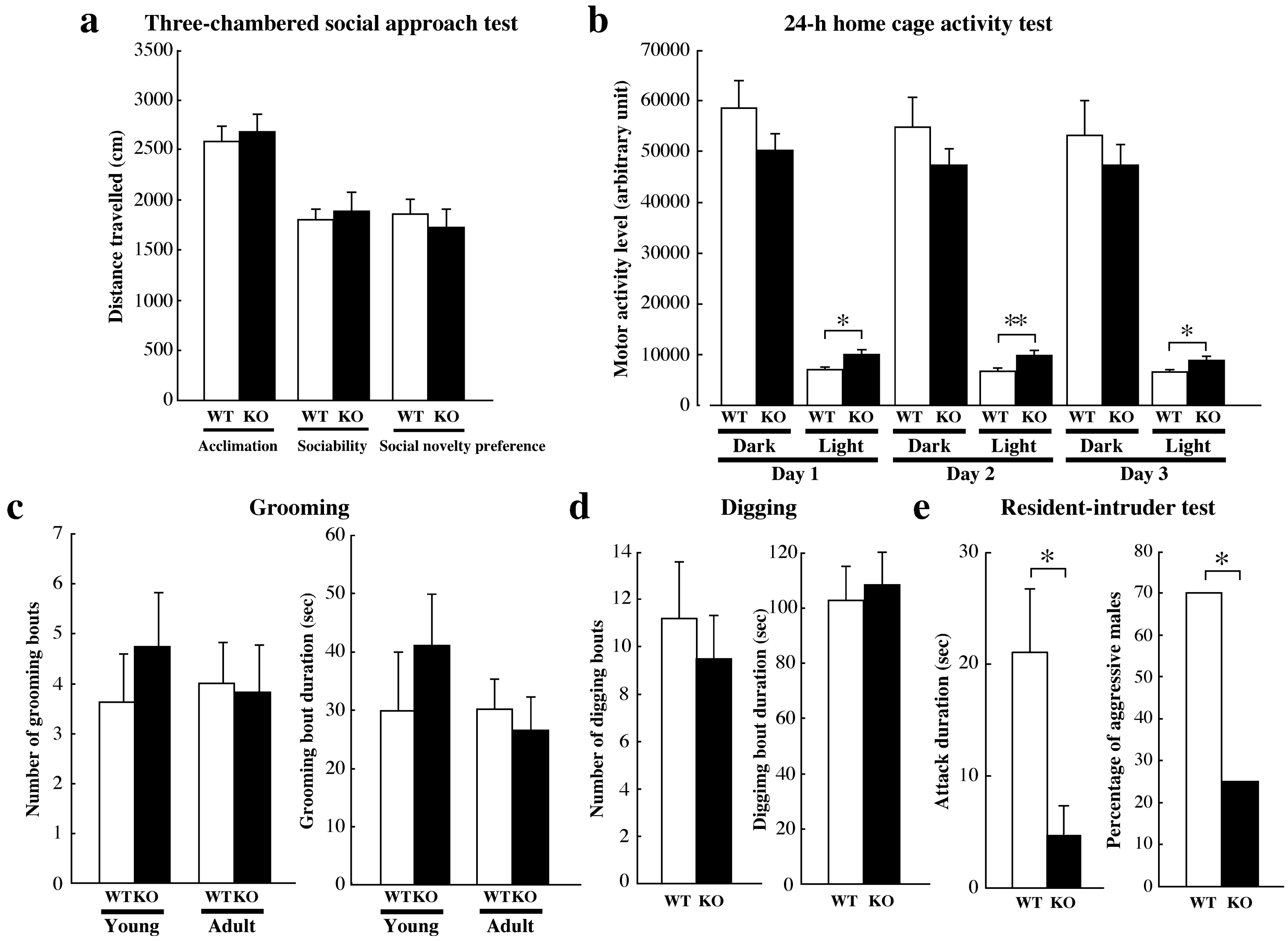
a
Three-chambered social approach test
Distance travelled (cm)
3500
3000
2500
2000
1500
1000
500
0
WT KO
Acclimation
WT KO
Sociability
WT KO
Social novelty preference
b
24-h home cage activity test
Motor activity level (arbitrary unit)
70000
60000
50000
40000
30000
20000
10000
0
*
**
*
WT KO
Dark
WT KO
Light
Day 1
WT KO
Dark
WT KO
Light
Day 2
WT KO
Dark
WT KO
Light
Day 3
c
Grooming
Number of grooming bouts
7
6
5
4
3
2
1
0
WTKO
Young
WTKO
Adult
Grooming bout duration (sec)
60
50
40
30
20
10
0
WTKO
Young
WTKO
Adult
d
Digging
Number of digging bouts
14
12
10
8
6
4
2
0
WT KO
Digging bout duration (sec)
120
100
80
60
40
20
0
WT KO
e
Resident-intruder test
Attack duration (sec)
30
20
10
0
*
WT KO
Percentage of aggressive males
80
70
60
50
40
30
20
10
0
*
WT KO

Supplementary Figure S3. Normal locomotor activity under dark conditions and normal grooming and digging behaviours of Kirrel3$^{-/-}$ mice in the home cage.

(a) The three-chambered social approach test. There were no significant differences in the distance travelled throughout experiment between wild-type (WT) and Kirrel3$^{-/-}$ (KO) mice. (b) The 24-h home cage activity test. Motor activity was not significantly different between wild-type (WT) and Kirrel3$^{-/-}$ (KO) mice during the dark phase (wild-type n = 15, Kirrel3$^{-/-}$ n = 22, $p = 0.26$; two-way repeated measures ANOVA), whereas the increase in motor activity was observed in Kirrel3$^{-/-}$ mice during the light phase ($p = 0.0047$; two-way repeated measures ANOVA with Bonferroni-Dunn *post hoc* test; $^{*}p < 0.05$, $^{**}p < 0.01$; one-way ANOVA with Tukey's *post hoc* test at the same sessions). (c, d) Grooming (c) and digging (d) behaviours in the home cage. The number of grooming (c, left panel) or digging (d, left panel) bouts and the duration of grooming (c, right panel) or digging (d, right panel) bouts were measured for 10 min. There were no significant differences in these behaviours between wild-type (WT) and Kirrel3$^{-/-}$ (KO) mice (young mice: wild-type n = 8, Kirrel3$^{-/-}$ n = 8, number of grooming bouts: $p = 0.41$; duration of grooming bouts: $p = 0.45$; adult mice: wild-type n = 6, Kirrel3$^{-/-}$ n = 6, number of grooming bouts: $p = 0.89$; duration of grooming bouts: $p = 0.64$; number of digging bouts: $p = 0.93$; duration of digging bouts: $p = 0.58$; one-way ANOVA). (e) The resident-intruder test. The attack duration (left panel, wild-type n = 10, Kirrel3$^{-/-}$ n = 12, $^{*}p < 0.05$; one-way ANOVA with Tukey's *post hoc* test) and the percentage of aggressive mice (right panel, $^{*}p < 0.05$; chi-square test) were significantly decreased in Kirrel3$^{-/-}$ (KO) mice compared with wild-type (WT) mice. Error bars indicate SEM.

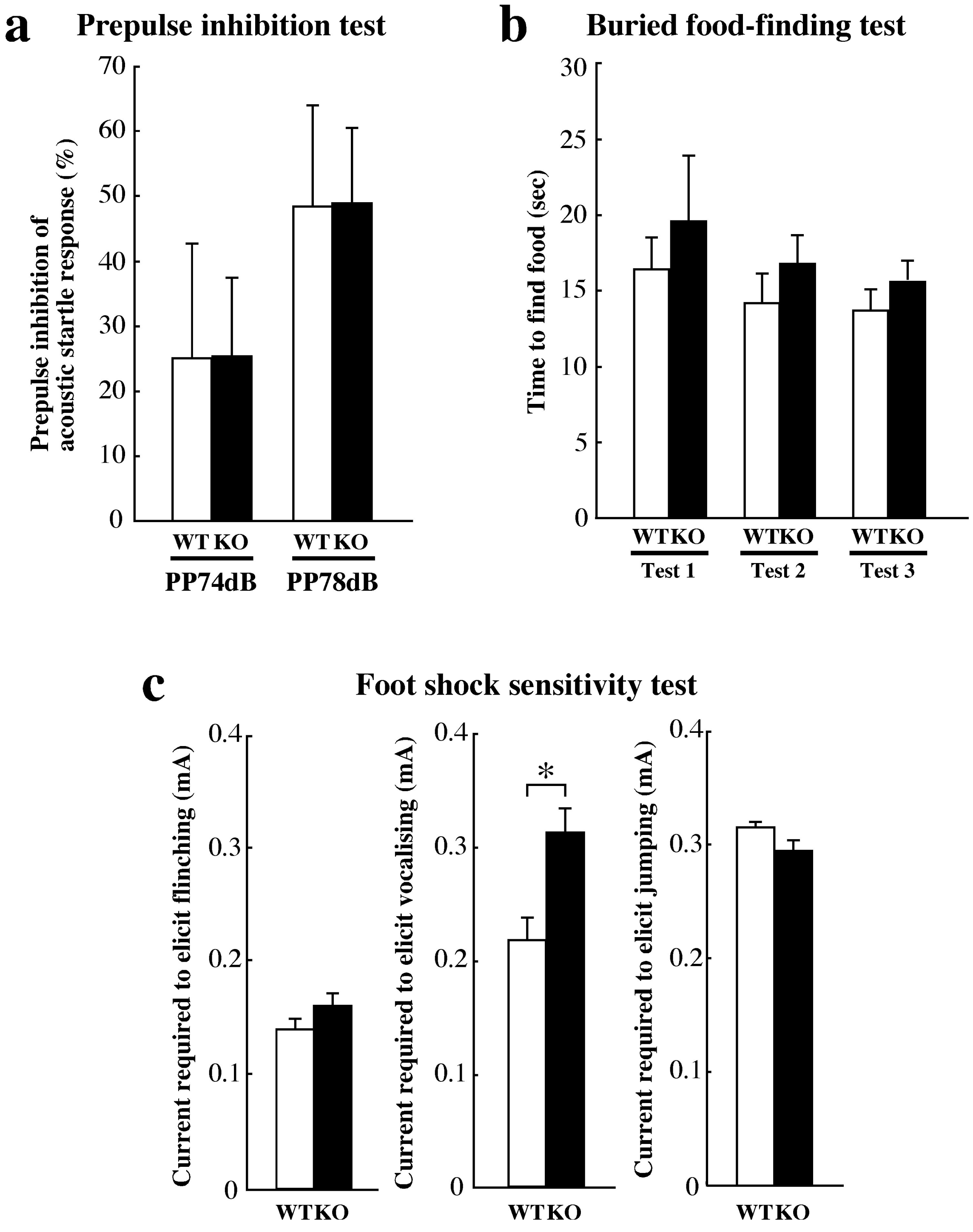
a
Prepulse inhibition test
Prepulse inhibition of acoustic startle response (%)
70
60
50
40
30
20
10
0
WT KO
WT KO
PP74dB
PP78dB
b
Buried food-finding test
Time to find food (sec)
30
25
20
15
10
5
0
WTKO
WTKO
WTKO
Test 1
Test 2
Test 3
c
Foot shock sensitivity test
Current required to elicit flinching (mA)
Current required to elicit vocalising (mA)
Current required to elicit jumping (mA)
0.4
0.3
0.2
0.1
0
*
WTKO
WTKO
WTKO

Supplementary Figure S4. Normal sensory-motor gating responses and olfactory sensitivity, but abnormal sensory responses to pain stimuli, in Kirrel3$^{-/-}$ mice. (a) In the PPI test, there were no significant differences between wild-type (WT) and Kirrel3$^{-/-}$ (KO) mice (wild-type n = 9, Kirrel3$^{-/-}$ n = 8, p = 0.94; two-way repeated measures ANOVA), indicating intact sensorimotor gating. (b) The buried food-finding test. There were no significant differences in the time to find the butter cookie between wild-type (WT) and Kirrel3$^{-/-}$ (KO) mice (wild-type n = 23, Kirrel3$^{-/-}$ n = 20, p = 0.19; two-way repeated measures ANOVA). (c) The foot shock sensitivity test. A higher current to elicit vocalisation (middle panel) was required in Kirrel3$^{-/-}$ (KO) mice compared with wild-type (WT) mice (wild-type n = 10, Kirrel3$^{-/-}$ n = 9, *p < 0.05; one-way ANOVA with Tukey's *post hoc* test). Error bars indicate SEM.

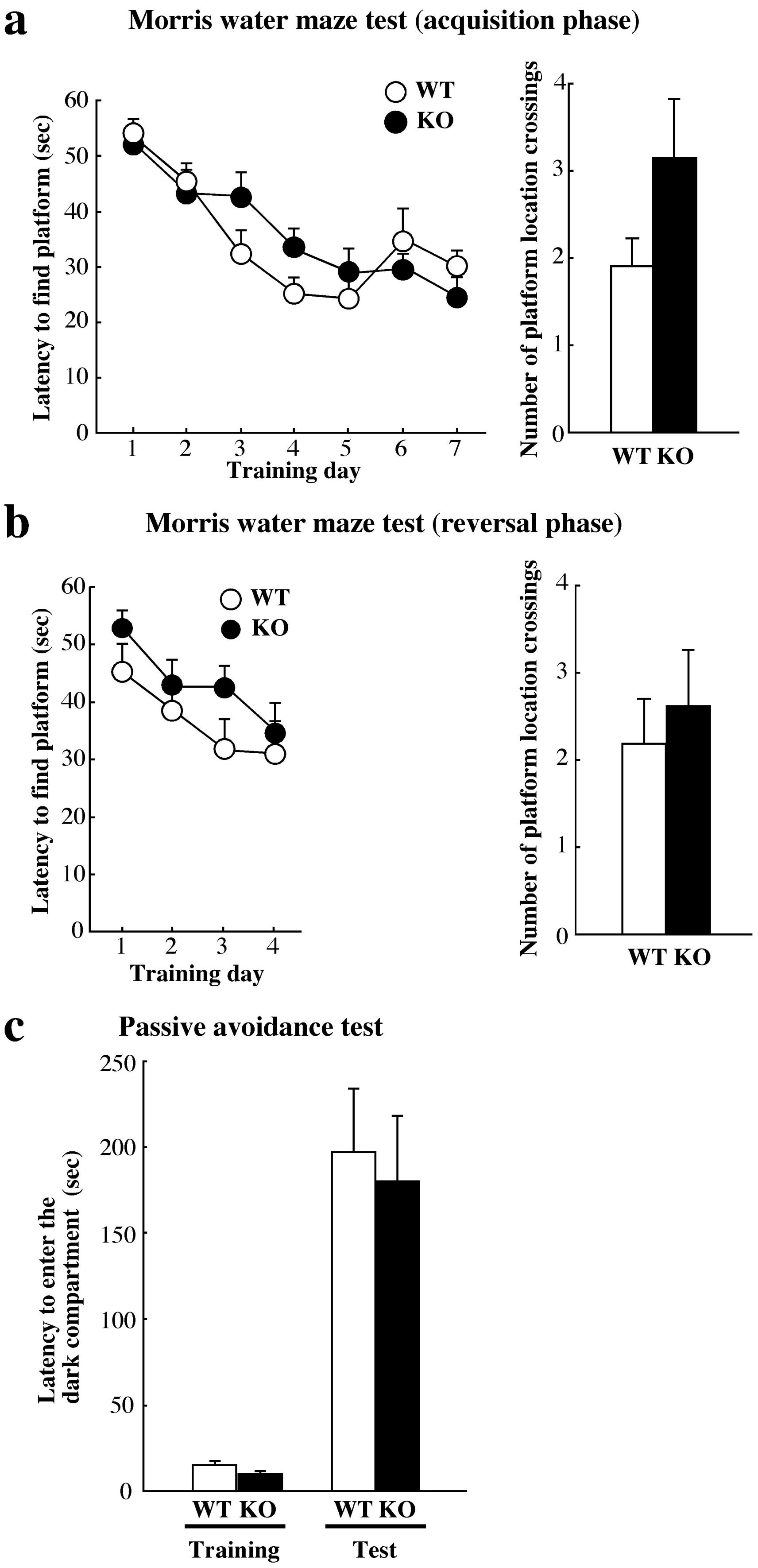
a
Morris water maze test (acquisition phase)
WT
KO
Latency to find platform (sec)
Training day
Number of platform location crossings
WT KO
b
Morris water maze test (reversal phase)
WT
KO
Latency to find platform (sec)
Training day
Number of platform location crossings
WT KO
c
Passive avoidance test
Latency to enter the dark compartment (sec)
WT KO
Training
WT KO
Test

Supplementary Figure S5. Normal spatial working memory and normal fear memory in Kirrel3$^{-/-}$ mice. (a) The Morris water maze test (acquisition phase). Both wild-type (WT) and Kirrel3$^{-/-}$ (KO) mice performed well at day 6 and 7 (left panel), indicating no impairment of spatial learning (wild-type n = 11, Kirrel3$^{-/-}$ n = 13, p = 0.64; two-way repeated measures ANOVA). In the probe test, there were no significant differences in the number of platform location crossings (right panel) between wild-type and Kirrel3$^{-/-}$ mice (p = 0.13; one-way ANOVA). (b) The Morris water maze test (reversal phase). No significant differences were observed in learning (wild-type n = 11, Kirrel3$^{-/-}$ n = 13, p = 0.16; two-way repeated measures ANOVA). In the probe test, there were no significant differences in the number of platform location crossings (right panel) between wild-type (WT) and Kirrel3$^{-/-}$ (KO) mice (p = 0.62; one-way ANOVA). (c) The passive avoidance test. The latency to enter the dark compartment 24 h after the foot shock was similar between wild-type (WT) and Kirrel3$^{-/-}$ (KO) mice (wild-type n = 7, Kirrel3$^{-/-}$ n = 11, p = 0.69; two-way repeated measures ANOVA), indicating intact fear memory in both genotypes. Error bars indicate SEM.

Supplementary Methods

Generation of Kirrel3$^{-/-}$ mice

The targeting construct was designed to replace the exon 1 of the Kirrel3 coding sequence containing a lacZ (β-galactosidase) reporter with a mouse nuclear localisation signal (NLS) upstream of a phosphoglycerine kinase (PGK) promoter driving a neomycin (Neo) selection marker. The PGK-Neo gene with polyadenylation site was flanked by loxP sites and could be later deleted by Cre recombinase (Cre). The deleted part of the Kirrel3 gene includes the entire Kirrel3 coding region starting with the translation initiation codon in exon 1 and ending downstream of exon 16, the last exon. The targeting construct was assembled from the Kirrel3 5' and 3' homology arms and NLS-lacZ restriction fragments, which were each amplified by PCR, subcloned into pPCR, and characterised by DNA sequencing. The 5' homology arm (4175 bp in length) was generated by PCR with the primers (5'-TGGCGCGCCCCACTGGGACCATAATAAGCCTGA and 5'-ACTGAAATCTCCACCTACGCCA). A *Spe*I site was introduced, which was later used in genomic screening of Southern blots using the 5' probe (underlined sequences annealed to the template). The 3' homology arm (6178 bp in length) was generated by PCR with the primers (5'-TGCGGCCGCGGCGCGCCGCTAGCTGAGCCATCAATGCTACCTGTAAG and 5'-TGCGGCCGCGCTGCCAAAACAACATCGTGAC). An NheI site was introduced, which was later used in genomic screening of Southern blots using the 3' probe (underlined sequences annealed to the template).

The final targeting construct was linearised and electroporated into embryonic stem (ES) cells, and colonies were selected for resistance to G418. Genomic DNA isolated

from ES cell colonies were digested with XbaI before hybridisation with 5' flanking probe. The wild-type allele produced a band of 6.8 kb, whereas the targeted allele produced a band of 9.7 kb. Mutant ES cell clones were injected into C57BL/6 blastocysts to produce chimeric mice. The chimeric mice were mated with C57BL/6 mice to obtain heterozygous mice carrying the targeted allele. Heterozygous offspring were mated with B6 Cre delete mice (Ozgene Pty Ltd) to remove the loxP-flanked selectable marker cassette. The founder mice were bred for more than 10 generations onto a C57BL/6J background. From cross-breeding of Kirrel3$^{+/-}$ mice, we obtained Kirrel3$^{-/-}$ and wild-type littermate mice for anatomical, histological and behavioural analyses.

Genotyping was performed by PCR of genomic DNA obtained by tail biopsy. Primer F3 (GATGCCATGGGTACAGAAAG) and primer R1 (CTCCTTACAGGTAGCATTG) amplified a 0.8 kb fragment in the wild-type allele, whereas primer F1 (GATTTGGTGGCGACGACTCCT) and primer R1 amplified a 0.43 kb fragment from the mutant allele (see Supplementary Fig. S1a). The PCR conditions were 94°C for 3 min; 30 cycles of 94°C for 30 seconds (sec), 60°C for 30 sec, 72°C for 1 min, followed by incubation at 72°C for 7 min.

Western blot analysis

Western blot analysis was performed as described previously (64) with some modifications. Briefly, 10-week-old wild-type and Kirrel3$^{-/-}$ mice were deeply anaesthetised with isoflurane, and the brains were quickly removed. The olfactory bulb, hippocampus, and cerebellum were dissected and homogenised in RIPA buffer (50 mM Tris-HCl, pH 7.4, 150 mM NaCl, 0.25% deoxycholic acid, 1% Nonidet P-40,

and 1 mM ethylenediaminetetraacetic acid; Upstate Biotechnology, Lake Placid, NY) containing protease inhibitor cocktail (Millipore, Temecula, CA), 2 mM sodium orthovanadate, 1 mM sodium fluoride, and 1 mM phenylmethylsulfonyl fluoride. The protein concentrations were measured with a BCA Protein Assay kit (Pierce Biotechnology, Rockford, IL). Twenty-five micrograms of protein was added to the sample buffer (125 mM Tris-HCl, pH 6.8, 4% sodium dodecyl sulphate [SDS], 20% glycerol, 0.002% bromophenol blue, and 10% 2-mercaptoethanol; Wako Chemical, Osaka, Japan) and separated with SDS-polyacrylamide gel electrophoresis, then transferred to the nitrocellulose membrane (GE Healthcare, Tokyo, Japan). The blotted membranes were blocked in 5% ECL blocking agent (GE Healthcare) for 1 h at room temperature (RT), then incubated with sheep anti-Kirrel3 antibody (1:200; R & D Systems, Minneapolis, MN) for 16 h at 4°C. After washing three times for 5 min, the membranes were incubated with a peroxidase-conjugated donkey anti-sheep immunoglobulin G (IgG) antibody (1:20000; Jackson ImmunoResearch Laboratories, West Grove, PA) for 1 h at RT. Immunoreaction was visualised with chemiluminescence using ECL detection reagent (GE Healthcare) according to the manufacturer's instructions. After the blotted membranes were stripped in 0.25 M glycine, pH 2.5 for 10 min at RT, they were incubated with mouse anti-β-actin antibody (1:10000; Sigma, St. Louis, MO) for 16 h at 4°C, followed by incubation with horseradish peroxidase-conjugated sheep anti-mouse IgG antibody (1:10000; GE Healthcare) for 1 h at RT.

Tissue preparation and haematoxylin-eosin staining

Under deep anaesthesia with isoflurane, 10- to 12-week-old wild-type and Kirrel3$^{-/-}$

mice were transcardially perfused with saline, followed by ice-cold 0.1 M phosphate-buffered saline (pH 7.4) containing 2% paraformaldehyde and 0.2% picric acid (Zamboni's fixative). The brains were quickly removed, sectioned sagittally or coronally, and immersed in the same fixative for 3 h at 4ºC. The specimens were cryoprotected in 0.1 M phosphate buffer containing 20% sucrose for 16 h at 4ºC. They were embedded in optimal cutting temperature compound (Sakura Finetek, Tokyo, Japan), frozen rapidly in cold n-hexane on dry ice, and stored at −80ºC. Six-micrometre-thick frozen sections were stained with haematoxylin for 5 min and eosin for 3 min.

Behavioural tests

Twenty-four-hour home cage activity test

After acclimation to the home cage with fresh bedding for 3 days, locomotor activity was measured for 3 days with an activity-monitoring system (Supermex; Muromachi Kikai).

Repetitive behaviours

Grooming, digging, and rearing were observed to examine repetitive behaviours as described previously (39). Each test mouse was habituated in the home cage with fresh bedding for 10 min. Behaviour was then video-recorded for 10 min. The number of grooming, digging, or rearing bouts were counted manually and the durations of grooming or digging bouts were measured manually with a stopwatch.

Resident-intruder test

To evaluate male-male aggression, which is evoked by chemical cues in male urine, the resident-intruder test was performed as previously described (20) with some modifications. Resident male wild-type and Kirrel3$^{-/-}$ mice were individually housed for at least 2 weeks to establish the territory in the home cage. Eight-week-old C57/BL6J male mice housed in groups (3–5 mice per cage) were castrated and used as intruders. An intruder mouse was introduced into the resident home cage for a 10-min confrontation, then removed. After 24 h, the same intruder mouse was swabbed with urine freshly collected from sexually mature C57BL/6J male mice on the back and urogenital region, and introduced into the resident home cage for a 10-min confrontation. The behaviours were video-recorded and the attack duration was measured manually with a stopwatch.

Visual placing test

To evaluate visual acuity, the visual placing test was performed as described previously (65). Briefly, each test mouse was held by its tail 30 cm above the surface of a table and slowly lowered to the table. If mice have intact vision, they typically reach out the forelimbs to the table when they approach the table surface.

Buried food-finding test

The buried food-finding test was performed as described previously (42, 66). Starting 2 days prior to the test, mice received an unfamiliar butter cookie (Morinaga, Tokyo, Japan) with water *ad libitum*. After food deprivation for 12 h, each test mouse was placed in a clear cage containing 3 cm of fresh bedding and acclimated for 5 min. The test mouse was then removed from the cage and a piece of the cookie (0.6 g) was

hidden beneath 1.5 cm of bedding at the corner of the cage. The test mouse was placed at the opposite corner of the buried cookie and allowed to explore until finding the cookie. The latency to hold the cookie in both paws was recorded. Mice were given three trials (tests 1–3) with intertrial intervals of 10 min.

Olfactory habituation/dishabituation test

To evaluate the ability to discriminate between different odours, including non-social odours and social odours, the olfactory habituation/dishabituation test was performed as described previously (34, 42, 66) with some modifications. Odour stimulants were delivered with a cotton-tipped swab, which was adhered with tape on each cage lid and placed 6 cm from the bottom of the cage. Mice sniffed, but did not contact the cotton-tipped swab between the wire bars of the cage lid. After 1 h of acclimation to a cotton-tipped swab without any odour stimulants, each test mouse was exposed to five different odour stimulants: distilled water, two non-social odours (almond odour and banana odour), and two social odours (female mouse urine). Almond and banana odours were made with almond (1:1000 dilution; Golden Kelly Patent Flavor, Osaka, Japan) and banana flavouring solutions (1:100 dilution; Golden Kelly Patent Flavor), respectively. Urine (1:100 dilution) obtained from different C57BL/6J female mice were used as social odours. Each odour stimulant (70 μl) was presented for 2 min in three consecutive trials with intertrial intervals of 1 min. The behaviour was video-recorded and the time spent sniffing the swab was measured manually with a stopwatch. Sniffing was scored when the distance between the nose of test mouse and the swab was 1 cm or shorter.

Foot shock sensitivity test

Pain sensitivity was assessed with the foot shock sensitivity test as described previously (67) with some modifications. Each test mouse was placed in the centre of a chamber (25 × 25 × 30 cm) with a metallic grid floor connected to a scrambling shocker device (NeuroScience idea, Osaka, Japan). After habituation for 2 min, the test mouse was given electrical foot shocks for 4 s. The current was 0.05 mA in the initial trial and increased by 0.05 mA every trial with intertrial intervals of 20 sec. An observer manually recorded the current required to elicit flinching, vocalising, and jumping.

Morris water maze test

The Morris water maze test was performed as described previously (34, 68) with some modifications. A circular pool (120 cm diameter) was filled with 23 ± 1°C of water to a depth of 25.5 cm, located in a room with a number of extramaze visual cues. The circular pool was divided into four equal quadrants. A circular escape platform (10 cm diameter) was submerged 1 cm below the surface of the water away from the wall of the pool and located in the centre of one of the four quadrants.

The test consisted of two phases: acquisition and reversal. In the acquisition phase, the hidden escape platform was located in the same location in the pool. Each test mouse was placed in a start position and allowed to swim until it reached the escape platform. If the mouse failed to reach the platform within 60 sec, they were subsequently guided to the platform and remained there for 15 sec before being removed. Mice were given four training trials per day, each with a different start position in a random order for 7 consecutive days. In the reversal phase, the hidden

platform was located in the opposite quadrant (reversal location). Mice were given four training trials per day with a new location of the escape platform for 4 consecutive days. At the end of the acquisition phase and the reversal phase, after the platform was removed from the pool, a probe test and a reversal probe test (free swim for 60 sec) were conducted to evaluate spatial reference memory. The latency to reach the platform and the number of platform location crossings were recorded with a computerised video-tracking system (CompACT VAS system).

Passive avoidance test

To evaluate emotional learning and short-term memory, we performed the passive avoidance test as described previously (69) with some modifications. A passive avoidance box (NeuroScience idea) consisted of two components with a metallic grid floor, which were a small white illuminated compartment (9 × 10 × 20 cm, 600 lux) and a large black dark compartment (25 × 25 × 30 cm, 90 lux), separated by a sliding guillotine door (5 × 5 cm). The metallic grid floor in the dark compartment was connected to the scrambling shocker. The test consisted of two phases: acquisition and retention.

For the acquisition phase, each test mouse was placed into the light compartment and the sliding guillotine door was raised. When the mouse stepped through the gate and completely entered the dark compartment, the door was shut and an electrical foot shock (0.4 mA) was delivered for 4 sec through the grid floor via the scrambling shocker. Mice were immediately removed from the dark compartment and left in the home cage. For the retention phase (24 h after the acquisition phase), each test mouse was placed back into the light compartment, and the latency to step through the gate and

enter the dark compartment was measured up to 300 sec.

Rotarod test

Motor coordination and motor learning were assessed using an accelerating rotarod apparatus (UGO Basile, VA, Italy) as described previously (70) with some modifications. Each test mouse was placed on a rotating drum (3 cm diameter) for a maximum of 5 min, and the latency to fall from the rotating drum was measured. The speed of the rotating drum increased from 4 to 40 rpm over a 5-min period. Mice were given three training trials (trainings 1–3) with intertrial intervals of 30 min on the first day and one test trial on the second day.

Supplementary References

64. Komori, T. *et al*. Expression of kin of irregular chiasm-like 3/mKirre in proprioceptive neurons of the dorsal root ganglia and its interaction with nephrin in muscle spindle. J. Comp. Neurol. 511, 92-108 (2008).
65. Sadakata, T. *et al*. Autistic-like phenotypes in Cadps2-knockout mice and aberrant CADPS2 splicing in autistic patients. J. Clin. Invest. 117, 931-943 (2007).
66. Yang, M. *et al*. Low sociability in BTBR T + tf/J mice is independent of partner strain. Phisiol. Behav. 107, 649-662 (2012).
67. Blundell, J. *et al*. Neuroligin-1 deletion results in impaired spatial memory and increased repetitive behavior. J. Neurosci. 30, 2115-2129 (2010).
68. Vorhees, C. V. & Williams, M. T. Morris water maze: procedures for assessing spatial and related forms of learning and memory. Nat. Protoc. 1, 848-858 (2006).
69. Mazzucchelli, C. *et al*. Knockout of ERK1 MAP kinase enhances synaptic plasticity in the striatum and facilitates striatal-mediated learning and memory. Neuron 34, 807-820 (2002).
70. Kumakura, K. *et al*. Hyperactivity in novel environment with increased dopamine and impaired novelty preference in apoptosis signal-regulating kinase 1 (ASK1)-deficient mice. Neurosci. Res. 66, 313-320 (2010).
